# Implementing vision transformer for classifying 2D biomedical images

**DOI:** 10.1038/s41598-024-63094-9

**Published:** 2024-05-31

**Authors:** Arindam Halder, Sanghita Gharami, Priyangshu Sadhu, Pawan Kumar Singh, Marcin Woźniak, Muhammad Fazal Ijaz

**Affiliations:** 1 Department of Information Technology, Jadavpur University, Jadavpur University Salt Lake Campus, Plot No. 8, Salt Lake Bypass, LB Block, Sector III, Kolkata, West Bengal 700106 India; 2 Metharath University, 99, Moo 10, Bang Toei, Sam Khok, 12160 Pathum Thani Thailand; 3 Faculty of Applied Mathematics, Silesian University of Technology, Kaszubska 23, 44-100 Gliwice, Poland; 4 School of IT and Engineering, Melbourne Institute of Technology, Melbourne, 3000 Australia

**Keywords:** Biomedical image classification, Deep learning, Vision transformer, MedMNISTv2, BloodMNIST, BreastMNIST, PathMNIST, RetinaMNIST, Computational science, Computer science, Information technology

## Abstract

In recent years, the growth spurt of medical imaging data has led to the development of various machine learning algorithms for various healthcare applications. The MedMNISTv2 dataset, a comprehensive benchmark for 2D biomedical image classification, encompasses diverse medical imaging modalities such as Fundus Camera, Breast Ultrasound, Colon Pathology, Blood Cell Microscope etc. Highly accurate classifications performed on these datasets is crucial for identification of various diseases and determining the course of treatment. This research paper presents a comprehensive analysis of four subsets within the MedMNISTv2 dataset: BloodMNIST, BreastMNIST, PathMNIST and RetinaMNIST. Each of these selected datasets is of diverse data modalities and comes with various sample sizes, and have been selected to analyze the efficiency of the model against diverse data modalities. The study explores the idea of assessing the Vision Transformer Model’s ability to capture intricate patterns and features crucial for these medical image classification and thereby transcend the benchmark metrics substantially. The methodology includes pre-processing the input images which is followed by training the ViT-base-patch16-224 model on the mentioned datasets. The performance of the model is assessed using key metrices and by comparing the classification accuracies achieved with the benchmark accuracies. With the assistance of ViT, the new benchmarks achieved for BloodMNIST, BreastMNIST, PathMNIST and RetinaMNIST are 97.90%, 90.38%, 94.62% and 57%, respectively. The study highlights the promise of Vision transformer models in medical image analysis, preparing the way for their adoption and further exploration in healthcare applications, aiming to enhance diagnostic accuracy and assist medical professionals in clinical decision-making.

## Introduction

In the realm of medical image analysis, the intersection of artificial intelligence and healthcare has witnessed profound advancements^1–3^. Automation of biomedical image classification tasks reduces the workload on healthcare professionals, allowing them to focus on more complex aspects of patient care. Highly accurate biomedical image classification^2^ is useful for achieving high diagnostic accuracy and for reducing misdiagnosis and false positive cases. They aid in diagnosis, prognosis and treatment planning for patients. Highly accurate biomedical image classification can aid to detect diseases early by correctly identifying subtle abnormalities facilitating timely intervention and improving patient outcomes. They also support medical research and drug discovery as they help medical professionals to identify potential drug targets.

In comparison with general image classification tasks, biomedical image classifications face several challenges. Biomedical images can be highly complex with varying noise levels and artifacts, often requiring more specialized techniques for preprocessing. Biological structures exhibit significant variability both within the same class (intra-class variability) and between different classes (inter-class variability). The utilization of deep learning models has revolutionized the understanding of medical imaging data, fostering remarkable strides in diagnostic accuracy and treatment planning. Recent Convolutional Neural Network (CNN)^4^ advancements like ResNet^5^ models, DenseNet^6^ models etc. have made significant contributions in the field of biomedical image classification. They adaptively learn spatial hierarchies of features from input images and perform well in classification tasks. However, the locality bias of CNN models have made it hard to capture the long distance relationships in the images. Lately, Vision Transformer (ViT) model has emerged as efficient model to capture these long-distance relationships with the help of its self-attention mechanism, which allows the model to weigh the importance of different elements in the image patches based on the relationship to each other. This allows the ViT to capture global dependencies in data, making it beneficial for tasks requiring comprehensive analysis of complex biomedical images where considering the entire image is crucial to make accurate predictions.

**Figure 1:**
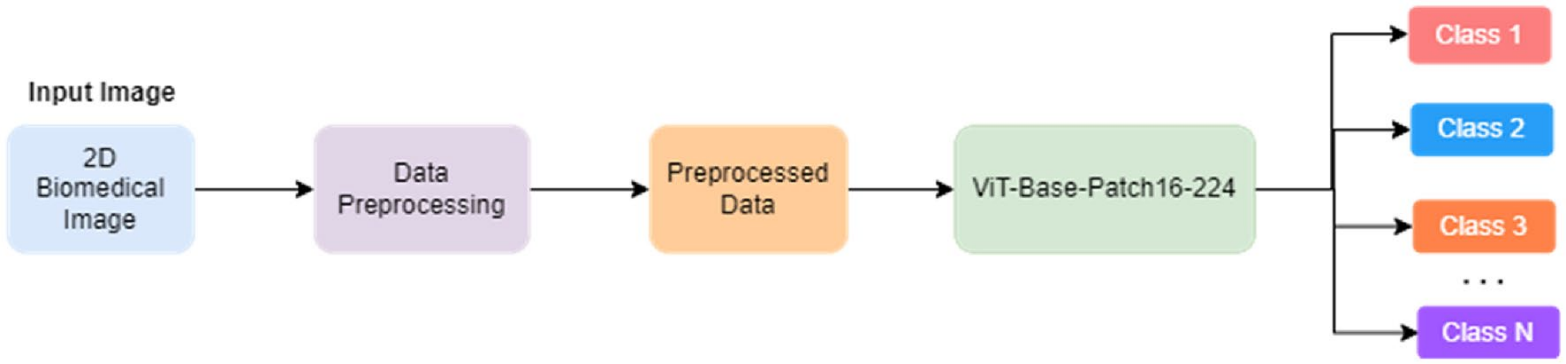
Block diagram of the proposed model pipeline for classifying 2D biomedical images.

The ViT^3^ architecture has emerged as a promising paradigm in various visual recognition tasks. However, in the field of biomedical image classification, the usage of CNN based models have been dominant. This study embarks on an exploration of the potential of ViT models in the context of biomedical image classification performed in various datasets of the MedMNISTv2^1^ collection. The MedMNISTv2 dataset encompasses diverse medical imaging modalities such as Fundus camera, Breast ultrasound, Colon pathology, Blood cell microscope etc. All the images are lightweight and pre-processed into 28 × 28. The study mainly focuses on using the ViT model to transcend the benchmark metrics of four datasets under MedMNISTv2: BloodMNIST, BreastMNIST, PathMNIST, RetinaMNIST. The outcomes of the experiments are benchmarked against the existing metrics showcasing superior performance and generalization capabilities of ViT models. The introduction of ViT models marks a departure from traditional convolutional neural networks (CNNs). But if trained on an insufficient amount of data, the ViT model may yield accuracies slightly less than CNN models like ResNet because of the absence of some of the inductive biases like local feature extraction and translation invariance. This problem is to overcome this issue by training ViT models on larger datasets. Hence, ViT models yield remarkable results if pre-trained extensively on large-scale data and then applied to tasks with limited data points^3^. Therefore, in this study we harness the power of a pre-trained ViT model, vit-base-patch-16-224^9^. Pre-trained on ImageNet-21k^7^ at 224 × 224 resolution and fine-tuned on ImageNet 2012^8^ dataset, vit-base-patch-16-224^9^ architecture splits the image into fixed-sized patches which are flattened and from these patches, lower-dimensional images are created. The self-attention mechanisms inherent in ViT architecture, enable them to capture intricate patterns and dependencies within the biomedical images. This is crucial for discerning subtle features and abnormalities and thus helps in accurate medical diagnosis. Figure 1 shows the schematic block diagram of our proposed model for the classification of 2D biomedical images.

In our study, we propose the following:

- Assess the ViT-Base-Patch16-224^9^’s ability to capture intricate patterns and features crucial for the biomedical image classification, which has been done for the first time, as per our knowledge.
- Present a comprehensive analysis of the four datasets of the MedMNISTv2 collection: BloodMNIST, BreastMNIST, PathMNIST and RetinaMNIST.
- Compare the accuracies achieved by our model for the four datasets against their benchmark accuracies and transcend them.
- Determine the efficiency of the model using other metrics in addition to the accuracy.
- By pushing the boundaries of performance on various datasets of MedMNISTv2 collection, the aim is to facilitate a more accurate and reliable biomedical image classification and analysis system, paving the way for improved diagnostic accuracy and treatment plan.

## Related works

In this section, we reviewed some of the works done in the field of biomedical image classification. Yang et al.^10^ developed MedMNIST^10^, which consists of ten medical medical datasets with 28 × 28 images, requiring no prior knowledge and is used for classification purposes. MedMNIST Classification Decathlon^5^ is designed to establish benchmarks of AutoML algorithms^11^ using the given datasets. It consists benchmark of several pre-trained deep learning algorithms like Res-Net50, Auto Keras^12^, Auto-sklearn on the 10 datasets namely ChestMNIST, PathMNIST, OCTMNIST, DermaMNIST, PneumoniaMNIST, BreastMNIST, RetinaMNIST, OrganMNIST A, OrganMNIST C, OrganMNIST S.

Liu et al.^13^ used FPViT for MedMNIST classification decathlon considering the respective limitations of it. FPViT that utilises a pyramid structure to make it an effective backbone for dense prediction tasks. The model efficiently integrates multi-scale feature maps from the foundational layers of ResNet^5^, creating a feature pyramid. FPViT combines both ResNet^5^ and ViT models to enhance feature learning and modelling. The model utilizes transformers to process the features derived from ResNet, viewing them as sequence data. This allows the model to capture long-range dependencies and global contexts within the feature maps, addressing the inherent limitations of convolution operations. FPViT uses multi-scale feature maps for enhanced classification and performance.

Lu et al.^14^ proposed adaptive conformal framework and incorporated it to address the issue of federated learning that has several challenges including suboptimal calibration and absence of clarity in interpretation which may hinder the broad acceptance of federated models in clinical areas. They applied this framework to the MedMNIST^10^ Medical Imaging benchmark, demonstrating enhanced coverage with lower average cardinality compared to local conformal predictions across six distinct benchmark datasets for medical imaging involving both 2-dimensional and 3-dimensional multi-class classification tasks. Conformal predictions are frameworks which are applicable to various models like quantile regression, decision trees and deep neural networks, establishing confidence sets based on the score function of the model.

Manzari et al.^15^ proposed challenges of CNN^4^ about the reliability in context to deep medical diagnosis systems explaining the vulnerability of these models to adversarial attacks. They addressed the potential problem of inaccurate diagnosis and proposed MedVit. MedVit is a highly robust combination of CNNs and Vision Transformer Model. It achieved notable resilience and generalization ability when compared with the state-of-the-art studies, particularly on a large-scale collection of standardized MedMNIST-2D datasets such as BloodMNIST, BreastMNIST, PathMNIST, RetinaMNIST with very less computational complexity.

Khan et al.^16^ proposed a method to address the potential problem of underfitting and overfitting of deep learning models and too big models respectively due to less availability of large medical datasets. They proposed Medi-CAT which combined several methodologies to mitigate the overfitting and underfitting problems in biomedical imaging datasets. The training incorporates large pre-trained vision transformers to address underfitting. To address overfitting it uses adversarial & contrastive learning techniques. It sees an increase of accuracy up to 2% on 4 datasets of MedMNIST i.e. OrganAMNIST, OrganCMNIST, OrganSMNIST, DermaMNIST.

Saha et al.^17^ introduced Isolated Federated Learning (IsoFed), a new learning scheme specifically designed for semi-supervised federated learning (SSFL). IsoFed addresses the issue that arises when some clients have entirely labelled data while others have completely unlabeled data, which is a typical scenario in medical images. IsoFed avoids the problem of combining supervised and semi-supervised models by learning them separately. Model performance was evaluated on MedMNIST datasets.

Herrmann et al.^18^ proposed a method Pyramid Adversarial Training (PyramidAT) to enhance the overall performance of ViT. Pyramid AT operates by perturbing input images across various scales while ensuring the perturbations at each scale remain constrained. This structure helps improve the attack’s effectiveness. It yields a 1.82% absolute enhancement in ImageNet^8^ clean accuracy when it was used to attack the ViTB model trained on only ImageNet-1K^19^ data.

Nguyen et al.^20^ introduced Self-Contrastively Supervised Learning (SelfCSL), a semi-supervised framework that leverages data from the same domain as the target task to train a pre-trained model via contrastive learning. This approach generates a pre-trained model with problem-specific features that leads to improved efficiency and stability. It used the MedMNIST^10^ dataset for testing and the method gives higher classification AUC^21^ compared to ImageNet^8^ in 5 out of 10 datasets and achieved greater stability in 9 out of 10 datasets.

Xu et al.^22^ addressed the vulnerability of deep neural networks to adversarial attacks, particularly in the medical domain where reliability is crucial. They observed that the existing defense methods effective for natural images were insufficient for medical images. Hence they proposed an easy-to-deploy and effective defence framework called MedRDF to counter adversarial attacks on medical pre-trained models. MedRDF generates multiple noisy copies of an image and obtains their output labels from the pre-trained model. It then applies majority voting to these labels to determine the final robust diagnostic result. They used DermaMNIST and COVID-19 datasets for verifying the effectiveness of MedRDF.

Yang et al.^1^ introduced MedMNISTv2, a comprehensive collection of MNIST-like datasets comprising standardized biomedical images. This dataset collection includes twelve datasets for 2D images and six datasets for 3D images. The images are resized to a compact format of 28 × 28 (for 2D) or 28 × 28 × 28 (for 3D) accompanied by the corresponding classification labels eliminating the need for background knowledge from users. MedMNISTv2 acts as an extension of the initial version, MedMNISTv1, featuring ten 2D datasets for biomedical image classification. While MedMNISTv1 has a more medical focus, v2 introduces two additional 2D biomedical image datasets. Recognizing the prevalence of 3D imaging in the biomedical area, MedMNISTv2 followed identical design patterns as those employed for the 2 dimensional biomedical image datasets and thoughtfully developed six 3D datasets.

### Motivation of our research

Our research is primarily inspired by the difficulties faced in biomedical image classification as it is a critical step in medical image analysis that uses different information to differentiate among different medical image datasets. Achieving the highest accuracy in medical image detection is important, since they are involved in diagnosing life threatening conditions. Various pre-trained CNN-based models such as ResNet-18, ResNet-50, Google AutoML Vision^33^ and many more have achieved some good accuracies in the MedMNISTv2^1^ dataset. Here, we have tried to enhance the overall classification accuracy much better to the best of our knowledge in various domains i.e., Blood, Breast, Path, Retina; each having its own set of challenges and complexities using ViT model as a tool. Reaching the highest classification accuracy is our goal and motivation, by which we can reduce the risk and challenges involved in biomedical image classification and increase its involvement and popularity in clinical and medical practices which may help clinicians evaluate medical images quickly, efficiently and with less error.

## Datasets utilized in our research

The dataset used in this study is the MedMNISTv2 dataset^1^, a lightweight benchmark for 2D and 3D biomedical image classification on a large scale. Within its comprehensive collection of twelve 2D and six 3D datasets, our study concentrates its efforts on four pivotal datasets: BloodMNIST, BreastMNIST, PathMNIST and RetinaMNIST.

**Figure 2:**
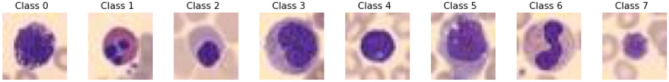
Various classes of BloodMNIST dataset.

**Figure 3:**
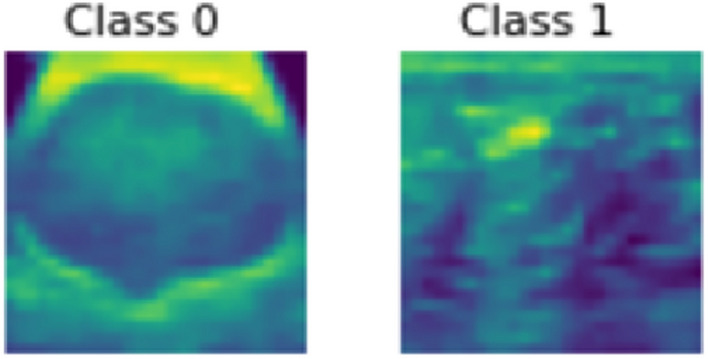
Various classes of BreastMNIST dataset.

**Figure 4:**
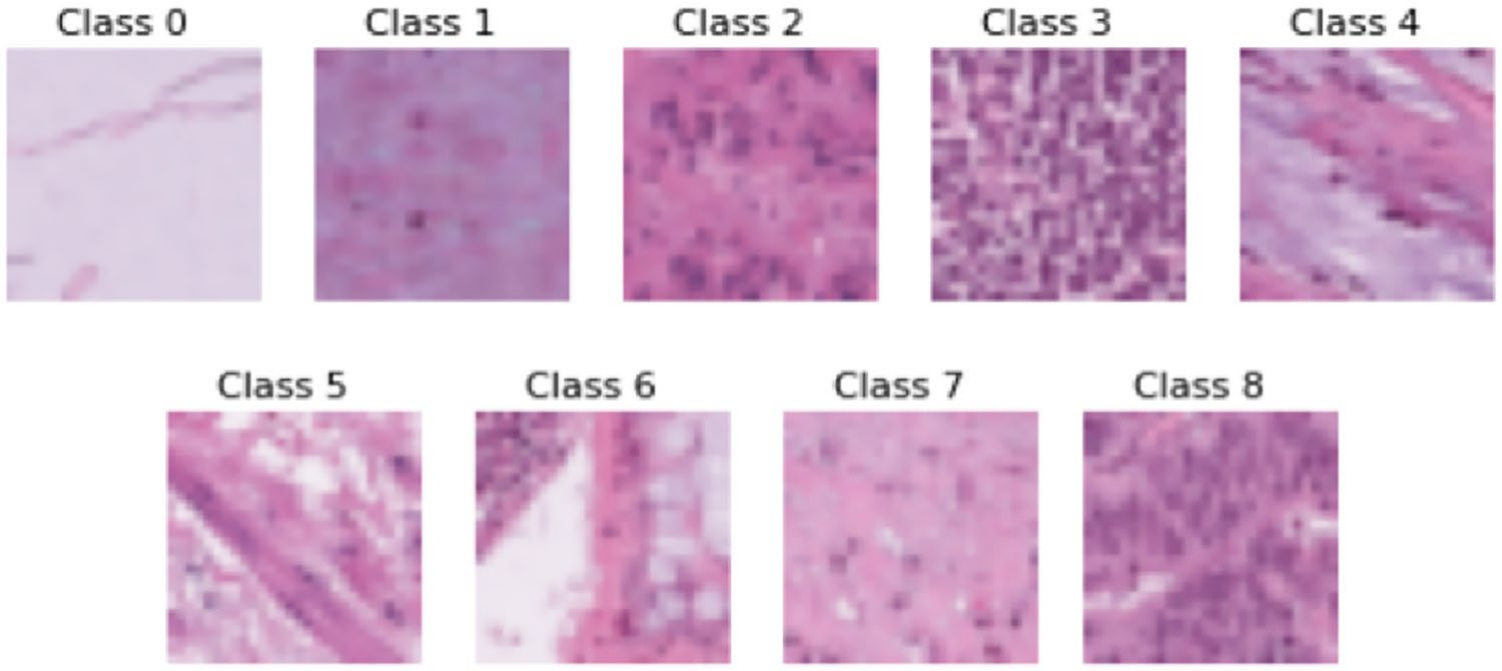
Various classes of PathMNIST dataset.

**Figure 5:**
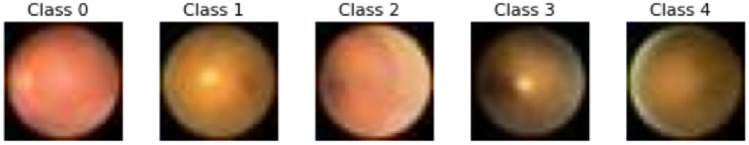
Various classes present in RetinaMNIST dataset.

- *BloodMNIST* dataset, based on a dataset^23,24^, comprising 17,092 meticulously curated images of individual normal blood cells from individuals free of infection, hematologic or oncologic disease, forms the cornerstone of our research. It is organized into 8 distinct classes. Figure 2 shows sample images from different classes in this dataset. The source dataset has been split into training, validation and test datasets at a ratio of 7:1:2.
- *BreastMNIST* dataset, originating from a repository of 780 breast ultrasound images^25^, stands as a binary classification dataset with three distinct classes: normal, benign and malignant. To reduce the classification problem into a binary classification, the dataset combines normal & benign classes as positive, juxtaposed against the malignant class as negative. Figure 3 shows sample images from each class of this dataset. The source dataset is divided into training, validation and test datasets at a ratio 7:1:2. Initially captured at a higher resolution of 1 × 500 × 500 pixels, later the images are resized to 1 × 28 × 28 pixels to streamline computational efficiency while preserving essential diagnostic features.
- *PathMNIST* dataset, based on a prior study^26,27^, derived from the extensive NCT-CRC-HE-100K^26,27^ dataset and augmented by the distinct CRC-VAL-HE-7 K test set^26,27^, represents a comprehensive collection of 100,000 image patches without overlap, derived from histological slides stained with hematoxylin and eosin while an additional 7,180 patches comes from a separate clinical center. This dataset encompasses 9 distinct tissue types pivotal in colorectal cancer assessment. This dataset offers a balanced representation for model training and validation by splitting the NCT-CRC-HE-100 K dataset into a 9:1 ratio for training and validation, while the CRC-VAL-HE-7 K acts like the test dataset. Figure 4 shows sample images of each of the eight classes present in this dataset.
- *RetinaMNIST* dataset, derived from the DeepDRID24^28^ challenge, encompasses 1,600 images of the retina fundus. The images are aimed at ordinal regression to grade diabetic retinopathy severity classification across 5 levels. Figure 5 shows sample images from five different classes found in this dataset. The source dataset is splitted into training and validation sets at a ratio 9:1 while repurposing the source validation set as test dataset. To streamline computational efficiency without compromising critical diagnostic features, the images, initially sized at 3 × 1736 × 1824 pixels are center-cropped and resized to a standardized 3 × 28 × 28 resolution.

## Methodology and implementation

### Data pre-processing

Data pre-processing is crucial for enhancing the quality of input data, eventually leading to efficient training of accurate models. In the MedMNISTv2^1^ collection, each dataset consisting of retinal images, pathology images, blood cell images, mammography images etc. presents unique challenges in biomedical image classification. All images in the MedMNISTv2 dataset are transformed into 28 × 28 with the classification labels correlated with it. To harness the power of transfer learning, we leveraged the pre-trained ViT model, specifically the *‘vit-base-patch16-224’* model from the Google Vision Transformer repository^29^. Transfer learning from a pre-trained model facilitates the extraction of generic features from different datasets, enhancing the ability of the model to recognize complex patterns in our specific biomedical image datasets.

**Figure 6:**
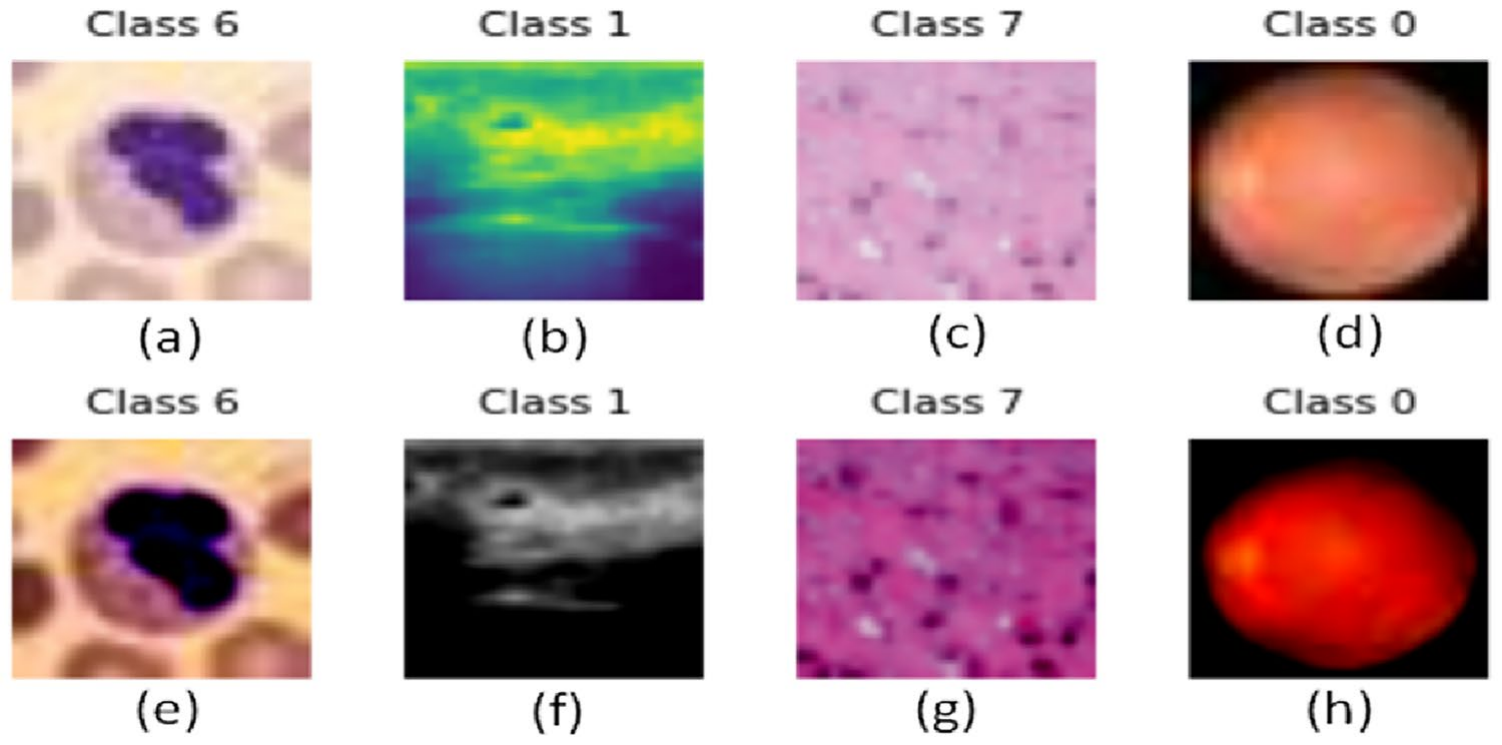
(**a**), (**b**), (**c**), (**d**) show the original images whereas (**e**), (**f**), (**g**), (**h**) illustrate its corresponding pre-processed images for BloodMNIST, BreastMNIST, PathMNIST, RetinaMNIST datasets respectively.

At first, the images are transformed into RGB format enabling the model to leverage color information. To ensure compatibility with the pre-trained ViT model, a standardized preprocessing pipeline is adopted. Using ViTImageProcessor, the images are resized to the model’s expected input size (224 × 224 pixels) and pixel values are normalized. After being pre-processed, the image shape is (3, 224, 224) and the label is a scalar. For seamless integration with the model, label encoding is conducted in target classes present in each dataset. Figure 6 illustrates samples of original as well as pre-processed images taken from each of the four datasets of MEDMNISTv2 collection.

### Proposed ViT architecture

In this research, the ViT architecture proposed by Dosovitskiy et al.^3^ is leveraged as the foundation for image classification tasks. The ViT model harnesses its self-attention mechanism to efficiently capture long distance relationships in data that enables it to grasp global dependencies. Self-attention mechanism allows the model to weigh the importance of different elements in the image patches based on the relationship to each other. It computes attention scores between every pair of patches, facilitating the understanding of how different parts of the image relate to each other. So instead of relying solely on local features, ViT captures the global dependencies which is beneficial for tasks like biomedical image classification where considering the entire image is crucial for accurate predictions. However due to the absence of some of the inductive biases like locality and translation invariance which are inherent in CNN models, ViT models may yield accuracies slightly less than some of the efficient CNN models if trained on insufficient data points. This problem can be overcome by training ViT models on large datasets. Hence ViT models yield remarkable results if pre-trained extensively on large-scale data and then applied to tasks with limited data points. So, in this study, a specific pre-trained ViT model provided by Google is utilized for the classification task i.e. “*vit-base-patch16-224”*. While fine-tuning this model, the weights learned from the ViT model, already pre-trained on a large dataset, are then further adjusted on a smaller dataset which is specific to the task at hand and demonstrate competitive performance compared to CNNs on various benchmarks.

#### The proposed technique

**Figure 7:**
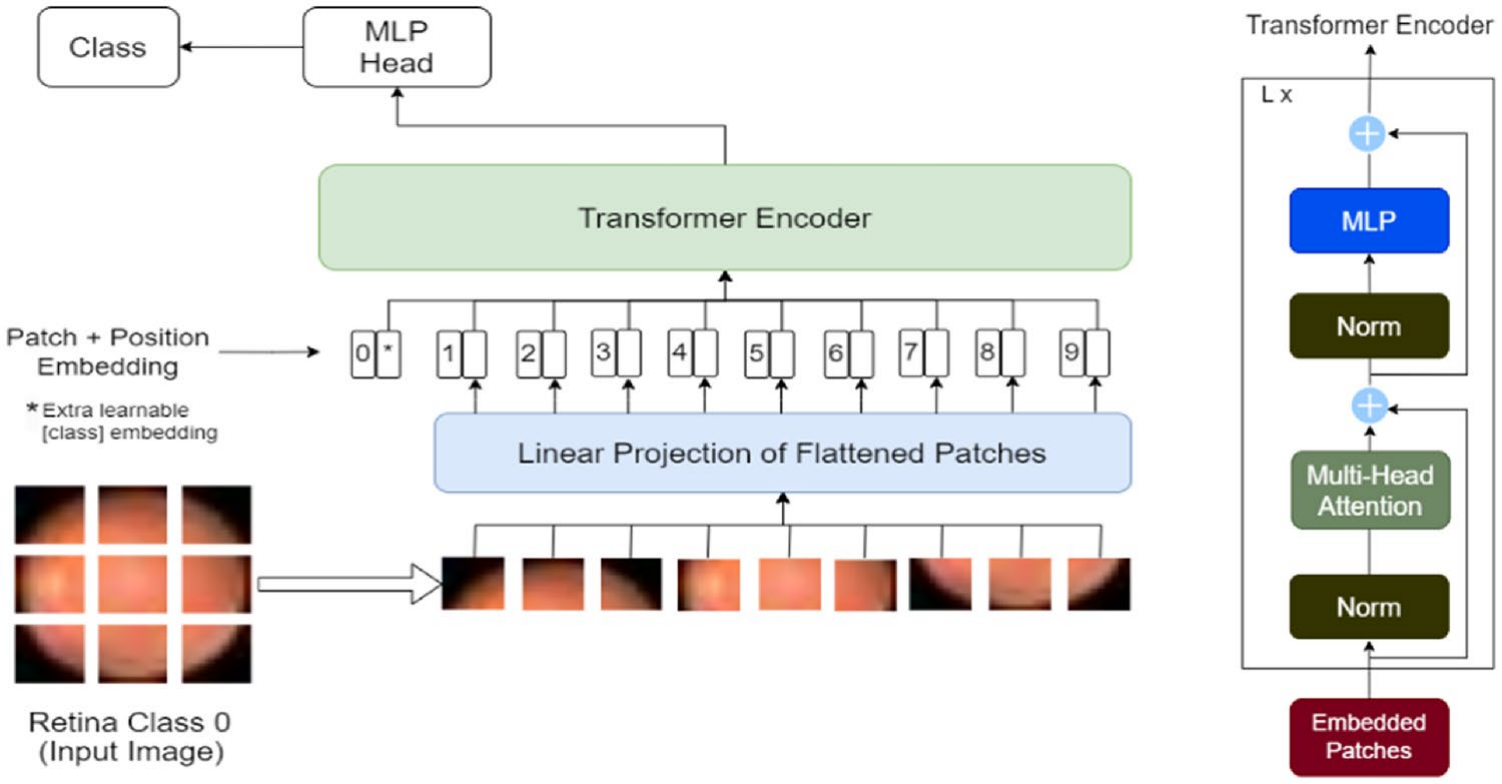
Architecture of the proposed ViT model.

The detail of our proposed ViT technique is illustrated in Fig. 7. We split the input image into fixed-size patches, each transformed into a vector through linear embedding. Position embeddings, crucial for spatial information, are then added to these embedded patches. This sequence of patches feeds into a standard transformer encoder which leverages self-attention mechanisms and captures intricate relationships within the image. A specialized “classification token” is introduced within the sequence to provide essential cues for the final classification decision-making.

#### ViT-Base-Patch16-224

ViT-Base-Patch16-224 ViT represents a groundbreaking fusion of Transformer architecture with computer vision. Initially pre-trained on the large dataset, ImageNet-21k^7^ and further fine-tuned on ImageNet^4^ (ILSVRC2012), ViT masters visual understanding through a sequence-based approach, converting images into fixed-size patches (16 × 16) from input images at a resolution of 224 × 224 and embedding them linearly. It introduces absolute position embeddings to retain spatial information i.e. crucial for transformer encoder’s layers. Comprising a stack of transformer encoder layers, this architecture alternates between multi-headed self-attention mechanisms and feedforward neural network^30^ blocks, bolstered by layer normalization and residual connections. By encapsulating the image information in patches and utilizing the transformer’s self-attention mechanisms, ViT-Base-Patch16-224 encapsulates a holistic understanding of images paving the way for effective image analysis tasks.

#### Key elements of ViT architecture

Two indispensable elements of the ViT architecture are the patch embedding projection and the class token projection. These two serve as a fundamental linear transformations, converting individual patches into distinct lower-dimensional vectors and amalgamating information from all patches into a reduced dimensional feature vector representing the entire image. Within the ViT architecture, the pivotal Multi-head Self-Attention (MSA) module transforms input vectors into query, key, and value vectors, providing a means to understand complex interdependencies within the image. Employing scaled dot-product attention, this module then calculates weighted sums of values based on attention weights, contributing to the overall comprehension of the image's intricate relationships. Layer normalization and residual connections contribute to stability in training by mitigating issues like vanishing gradient, ensuring efficient learning within deep networks. Additionally, the feedforward neural network^30^, consisting of linear layers and ReLU activation, converts MSA module outputs into feature vectors for individual patches, further reinforced by layer normalization stages for improved stability and efficacy in the ViT model’s operations. These components collectively empower ViT to process image patches, extract crucial spatial information and represent images comprehensively for diverse visual tasks.

#### Fine-tuning

In the typical pre-training process for ViTs, the model undergoes training on extensive datasets and subsequently fine-tunes on more specific downstream tasks. Here, we replace the pre-trained head with a zero-initialized D × K feedforward layer, tailored to the number of classes in the downstream task. In this segment, we utilized a pre-trained ViT model to push the boundaries of excellence in the realm of 2D biomedical image classification. We adopted an approach by utilizing the pre-trained *vit-base-patch16-224* model as the foundation of our work. We carefully fine-tuned the hyperparameters, such as batch size, learning rate, save steps, logging steps, evaluation steps, save total limit to guarantee effective convergence and generalization. With a learning rate of 5 × 10^–5^, we used the AdamW^31^ optimizer and defined the batch size per device as 32 for all the four datasets: BloodMNIST, BreastMNIST, PathMNIST & RetinaMNIST. For BloodMNIST dataset, save steps, logging steps and evaluation steps are set as 374. For the BreastMNIST dataset, save steps, logging steps and evaluation steps are set as 10. For the PathMNIST dataset, save steps, logging steps and evaluation steps are set as 200. For the RetinaMNIST dataset, save steps, logging steps and evaluation steps are set as 34. We conducted the training for 2 epochs on the PathMNIST dataset and for 10 epochs on the rest of the datasets. We defined the save total limit as 2 that defines that only the best two model checkpoints will be kept in our disk. Table 1 presents the general fine-tuning values set for all the four datasets whereas Table 2 defines the values of fine-tuning parameters that have been set for each of the four datasets: BloodMNIST, BreastMNIST, PathMNIST & RetinaMNIST.

**Table 1:** General values of fine-tuning parameters considered in the present work for all four datasets taken from MedMNISTv2.

Parameters	Batch_size (per device)	Learning_rate	Optimizer	Save_total_limit
Values	32	5 × 10^–5^	AdamW	2

**Table 2:** Fine-tuning parameters and their corresponding values for BloodMNIST, BreastMNIST, PathMNIST & RetinaMNIST datasets.

Parameters	BloodMNIST	BreastMNIST	PathMNIST	RetinaMNIST
save_steps	374	10	200	34
logging_steps	374	10	200	34
eval_steps	374	10	200	34
epochs	10	10	2	10

## Evaluation approach

In assessing the effectiveness of our proposed ViT model on the BloodMNIST, BreastMNIST, PathMNIST and RetinaMNIST datasets from the MedMNISTv2 collection, we utilized some key performance metrics which include Accuracy, F1 Score, Precision, Recall^32^ and confusion matrix. They provide a comprehensive evaluation of the model’s classification capabilities, addressing different aspects of classification correctness and balance between false positives and false negatives.

*Accuracy (ACC)* Accuracy is a foundational metric that represents the ratio of classes that are classified correctly out of all the predictions. It offers a global assessment of the model’s overall correctness.

1 $$ACC = (TP+TN) / (TP+TN+FP+FN)$$

*Precision* Precision refers to the measure of the accuracy of positive predictions. It represents the ratio of true positive predictions out of all instances predicted as positive.

2 $$Precision = TP / (TP + FP)$$

*Recall* Recall is the sensitivity of the model. It represents the ratio of true positively predicted instances out of all actual positive instances.

3 $$Recall = TP / (TP + FN)$$

*F1 Score* F1 Score is a balanced metric that considers both precision and recall. It helps us to understand the model’s trade-off between precision and recall.

4 $$F1 Score = 2*(\frac{\left(Precision*Recall\right)}{Precision+Recall})$$

*Confusion Matrix* Confusion Matrix gives a picture of the model’s predictions, providing the number of true positives, true negatives, false positives, false negatives for each class. This is valuable for understanding the nature of classification errors.

It is to be noted that all experimental protocols were approved by Department of Information Technology, Jadavpur University, Kolkata, India. To assess the efficiency of the model, we compared the accuracy of our ViT model against the benchmark accuracy on the BloodMNIST, BreastMNIST, PathMNIST and RetinaMNIST datasets. Our ViT model showed superior performance by transcending the benchmark accuracy for the mentioned datasets.

### Result analysis

*ViT-Base-Patch16-224* Vision Transformer model emerged as an efficient bench model for BloodMNIST, BreastMNIST, PathMNIST and RetinaMNIST dataset. We evaluated the efficiency of the models with metrics like accuracy, precision, recall, F1 Score^32^, Confusion Matrix. We compared the accuracy achieved by the model with the benchmark accuracies and transcended them. For the PathMNIST dataset, the epoch was set to be 2 and the model displayed a superior performance by achieving an accuracy of 94.62% and beating the benchmark accuracy of 91.1%. In case of the BloodMNIST and RetinaMNIST datasets, the epoch was set to be 10 and the models achieved the accuracies of 97.90% and 57.0% respectively beating the benchmark accuracies of 96.6% and 53.1% mentioned for the respective datasets. In the case of the BreastMNIST dataset, we initially evaluated the accuracy of the model by setting the epoch to be 3. The model achieved an accuracy of 86.4% for epoch = 3. For increasing the efficiency we set the epoch to be 10 for the dataset. For epoch = 10, the model achieved an accuracy of 90.38% beating the benchmark accuracy of 86.3%.

### Results on BloodMNIST

**Figure 8:**
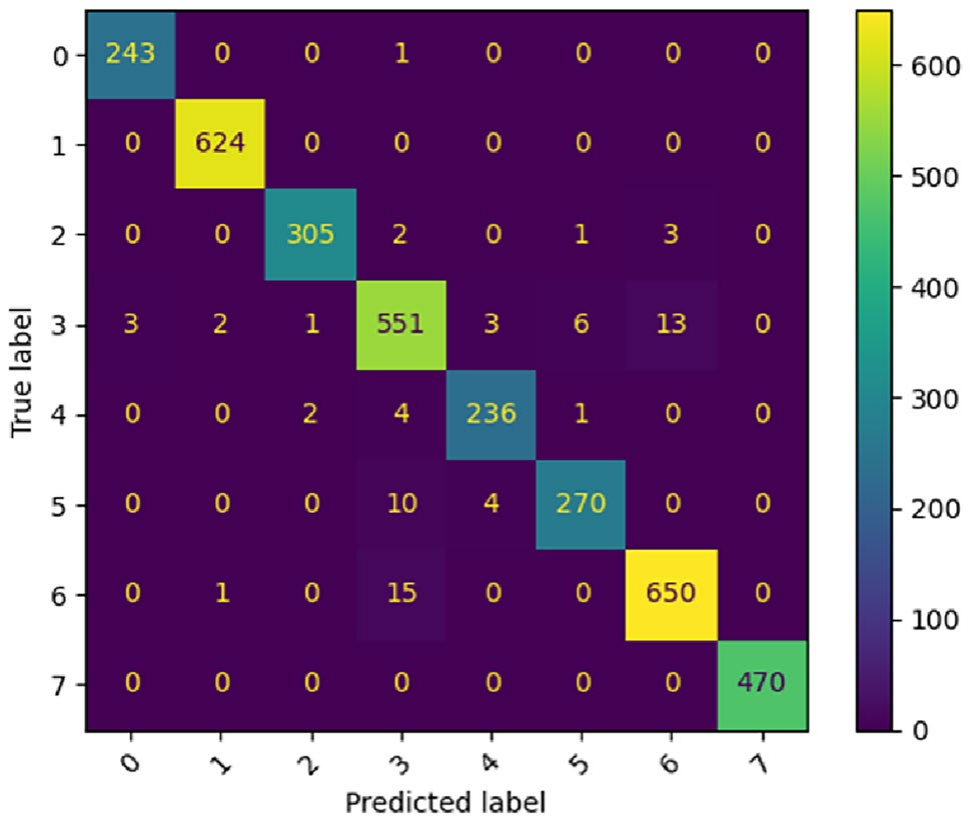
Confusion matrix produced by the proposed ViT model for BloodMNIST dataset.

Table 3 shows the detailed classification report on BloodMNIST dataset. The evaluation metrics such as F1-Score, Precision, Recall^32^, Support for each class and overall ACC have been considered to evaluate the performance of our proposed ViT model on the dataset. Figure 8 represents the confusion matrix for BloodMNIST dataset. The confusion matrix is valuable for understanding the nature of classification errors and thus helps in assessing the performance of the model against the mentioned datasets. The x-axis represents the predicted labels and the y-axis represents the actual labels.

**Table 3:** Classification report (in terms of Precision, Recall, F1-Score and Support) given by the proposed ViT model of each class present in BloodMNIST dataset.

Class label	Precision	Recall	F1-Score	Support
Basophil	0.99	1.00	1.00	244
Eosinophil	1.00	1.00	1.00	624
Erythroblast	0.99	0.98	0.99	311
Immature granulocytes (myelocytes, metamyelocytes and promyelocytes)	0.95	0.95	0.95	579
Lymphocyte	0.97	0.97	0.97	243
Monocyte	0.97	0.95	0.96	284
Neutrophil	0.98	0.98	0.98	666
Platelet	1.00	1.00	1.00	470
ACC	97.90%

In case of Fig. 8, for class ‘0’, the model correctly categorized 243 images as class ‘0’. The model misidentified one class ‘0’ image as class ‘3’ and no class ‘0’ image as rest of the classes. For class ‘1’, the model correctly categorized 624 images as class ‘1’. The model misidentified no class ‘1’ images. For class ‘2’, the model correctly categorized 305 images as class ‘2’. The model misidentified two class ‘2’ images as class ‘3’, one class ‘2’ image as class ‘5’, three class ‘2’ image as class ‘6’ and no class ‘2’ images as rest of the classes. For class ‘3’, the model correctly categorized 551 images as class ‘3’. The model misidentified three class ‘3’ images as class ‘0’, two class ‘3’ images as class ‘1’, one class ‘3’ image as class ‘2’, three class ‘3’ images as class ‘4’, six class ‘3’ images as class ‘5’ and thirteen class ‘3’ images as class ‘6’ and no class ‘3’ images as rest of the classes. For class ‘4’, the model correctly categorized 236 images as class ‘4’. The model misidentified two class ‘4’ images as class ‘2’, four class ‘4’ images as class ‘3’, one class ‘4’ image as class ‘5’ and no class ‘4’ images as rest of the classes. For class ‘5’, the model correctly categorized 270 images as class ‘5’. The model misidentified ten and four class ‘5’ images as class ‘3’ image and class ‘4’ image respectively and no class ‘5’ image as rest of the classes. For class ‘6’, the model correctly categorized 650 images as class ‘6’. The model misidentified one class ‘6’ image as class ‘1’, fifteen class ‘6’ images as class ‘3’ and no class ‘6’ images as rest of the classes. For class ‘7’, the model correctly categorized 470 images as class ‘7’ with no misidentified class ‘7’ images.

**Figure 9:**
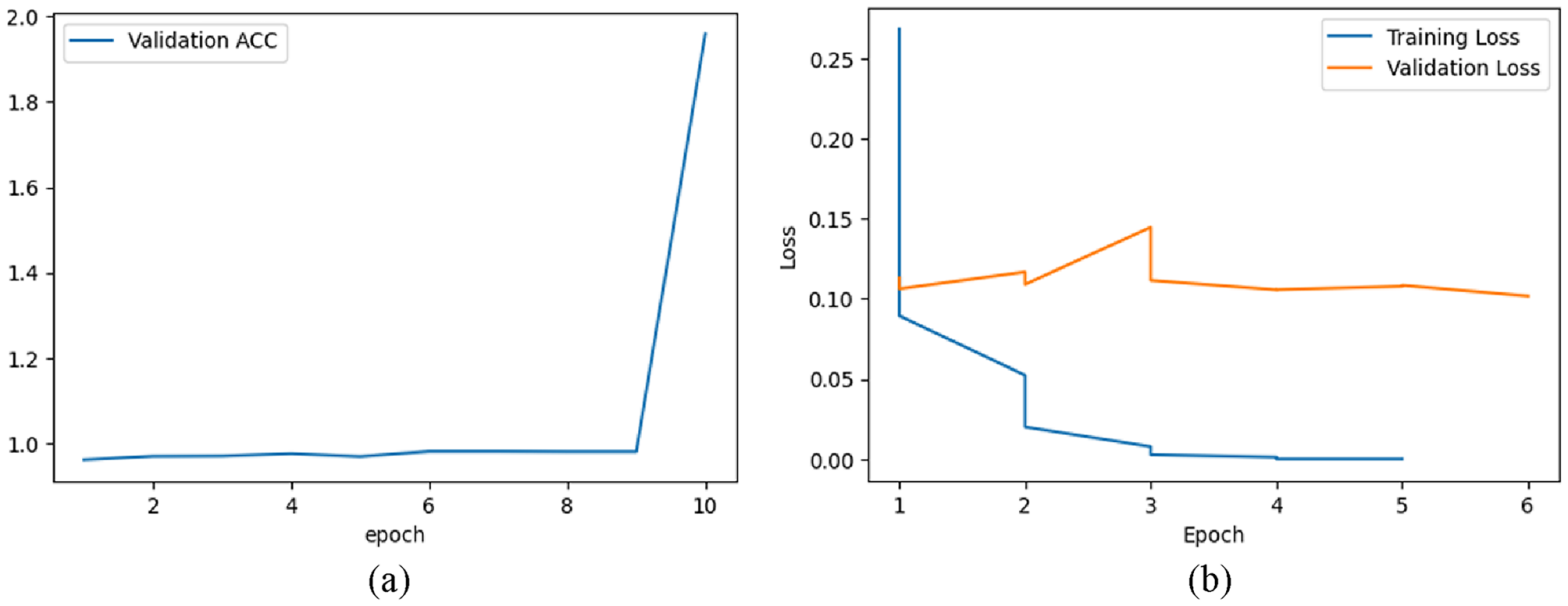
(**a**) Validation accuracy & (**b**) Train-validation loss graph for BloodMNIST dataset.

Figure 9a represents the validation accuracy curve for the BloodMNIST dataset over 10 epochs. It is a visual representation of how the accuracy of a model changes over the course of training when evaluated on a separate validation dataset. The x-axis represents the number of epochs while the y-axis represents the validation accuracy achieved by the model at each epoch. In Fig. 9a, at around epoch = 9, the accuracy curve begins a steep increase and around epoch = 10, it increases up to the accuracy value around 1.99.

Figure 9b shows the training and validation loss of the model on BloodMNIST dataset over six epochs. The training loss is lower than the validation loss. The training loss decreases over time as the model learns to fit the training data better. The validation loss increases up to around 0.15 till around epoch = 3. From around epoch = 3, the validation loss begins to decrease and at epoch = 6, its value is around 0.10.

**Figure 10:**
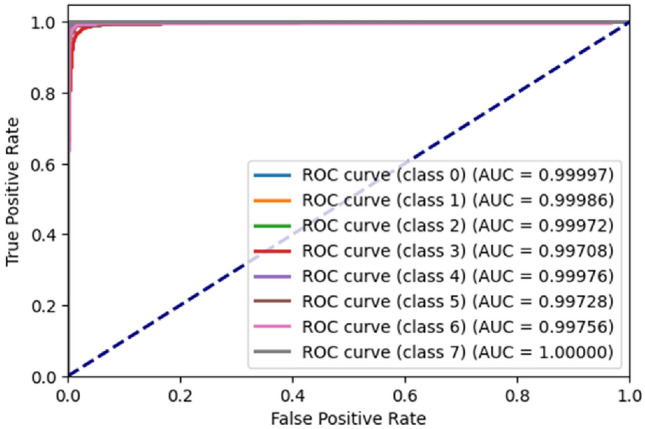
ROC curve generated by the proposed ViT model for BloodMNIST dataset.

Figure 10 represents the ROC^21^ Curve for BloodMNIST dataset. The x-axis represents the false positive rate while the y-axis represents the true positive rate. For class ‘0’, the AUC^21^ is 0.99997. For class ‘1’, the AUC is 0.99986. For class ‘2’, the AUC is 0.99972. For class ‘3’, the AUC is 0.99708. For class ‘4’, the AUC is 0.99976. For class ‘5’, the AUC is 0.99728. For class ‘6’, the AUC is 0.99756. For class ‘7’, the AUC is 1.00000 as no class ‘7’ image has been misclassified.

### Results on BreastMNIST

**Figure 11:**
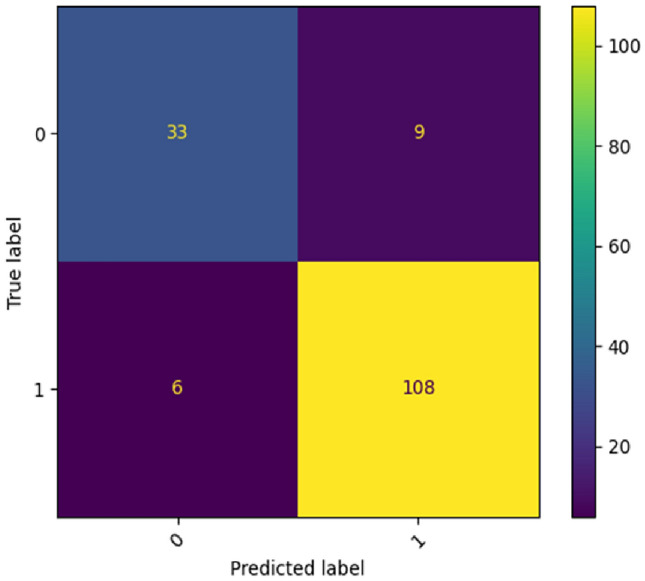
Confusion matrix produced by the proposed ViT model for BreastMNIST dataset.

Table 4 shows the detailed classification report on the BreastMNIST dataset. Figure 11 represents the confusion matrix for the BreastMNIST dataset.

**Table 4:** Classification report (in terms of Precision, Recall, F1-Score and Support) given by the proposed ViT model of each class present in BreastMNIST dataset.

Class label	Precision	Recall	F1-Score	Support
Malignant	0.85	0.79	0.81	42
Normal, benign	0.92	0.95	0.94	114
ACC	90.38%

In the case of Fig. 11, for class ‘0’, the model correctly categorized 33 images as class ‘0’. The model misclassified nine class ‘0’ images as class ‘1’. For class ‘1’, the model correctly categorized 108 images as class ‘1’. The model misidentified six class ‘1’ images as class ‘0’.

**Figure 12:**
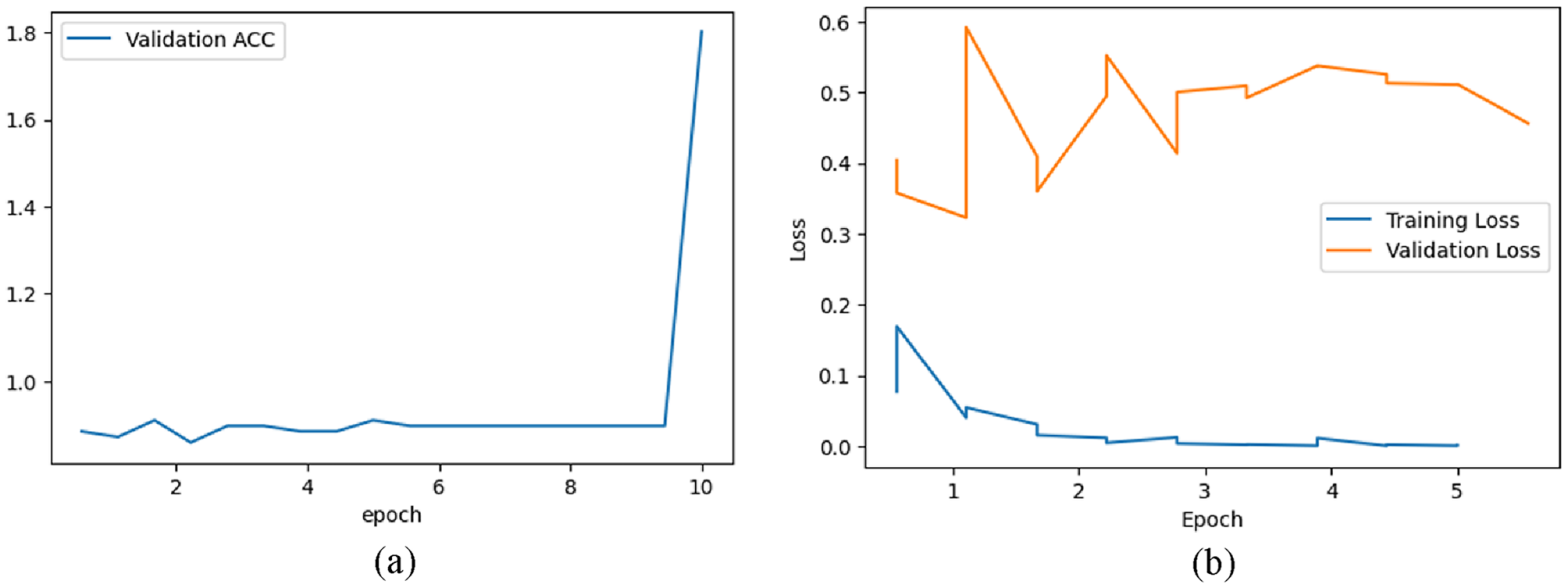
(**a**) Validation Accuracy & (**b**) Train-Validation Loss graph for BreastMNIST dataset.

Figure 12a represents the validation accuracy curve for the BreastMNIST dataset over ten epochs. In Fig. 12a, at around epoch = 2, the accuracy drops slightly before increasing. Between epoch = 9 and epoch = 10, the curve steeply increases and at epoch = 10, it increases up to the accuracy value around 1.8. Figure 12b shows the training and validation loss of the model on the BreastMNIST dataset over 5 epochs. The training loss is lower than the validation loss. The training loss decreases over time. The initial validation loss is around 0.4 which decreases until around epoch = 1. Between epoch = 1 and epoch = 2, the validation loss peaks at around 0.6 before decreasing to around 0.36 slightly before epoch = 2. From epoch = 2 to epoch = 5, there is an increase in validation loss from around 0.4 to around 0.5 with fluctuations in between. Overall, from epoch = 0 to epoch = 5, the validation loss has increased from around 0.4 to around 0.5.

**Figure 13:**
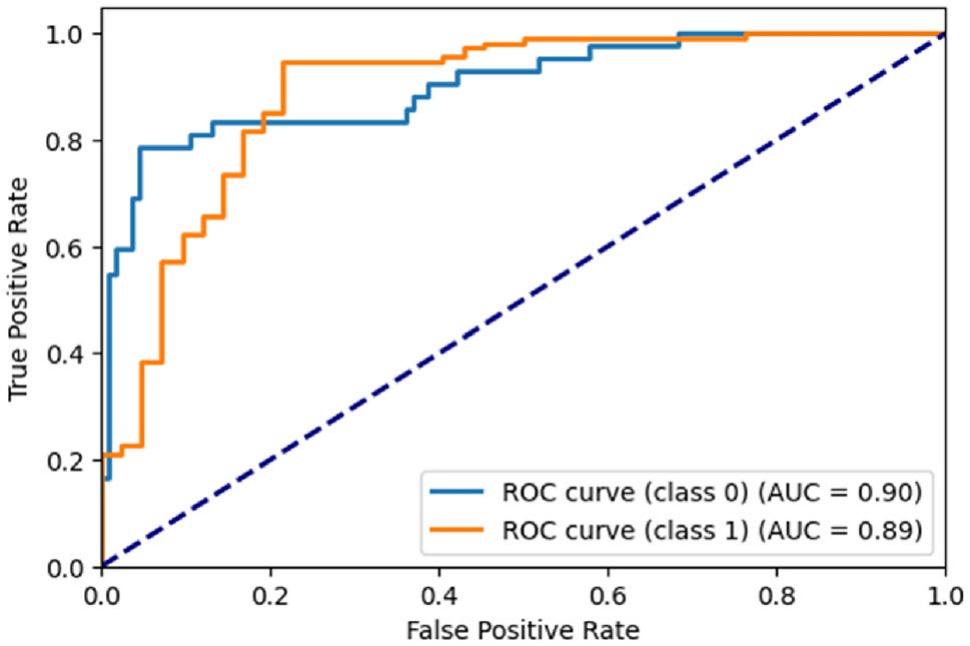
ROC curve generated by the proposed ViT model for BreastMNIST dataset.

Figure 13 represents the ROC^21^ Curve for BreastMNIST dataset. The x-axis represents the false positive rate while the y-axis represents the true positive rate. For class ‘0’, the AUC^21^ is 0.90. For class ‘1’, the AUC is 0.89.

### Results on PathMNIST

**Figure 14:**
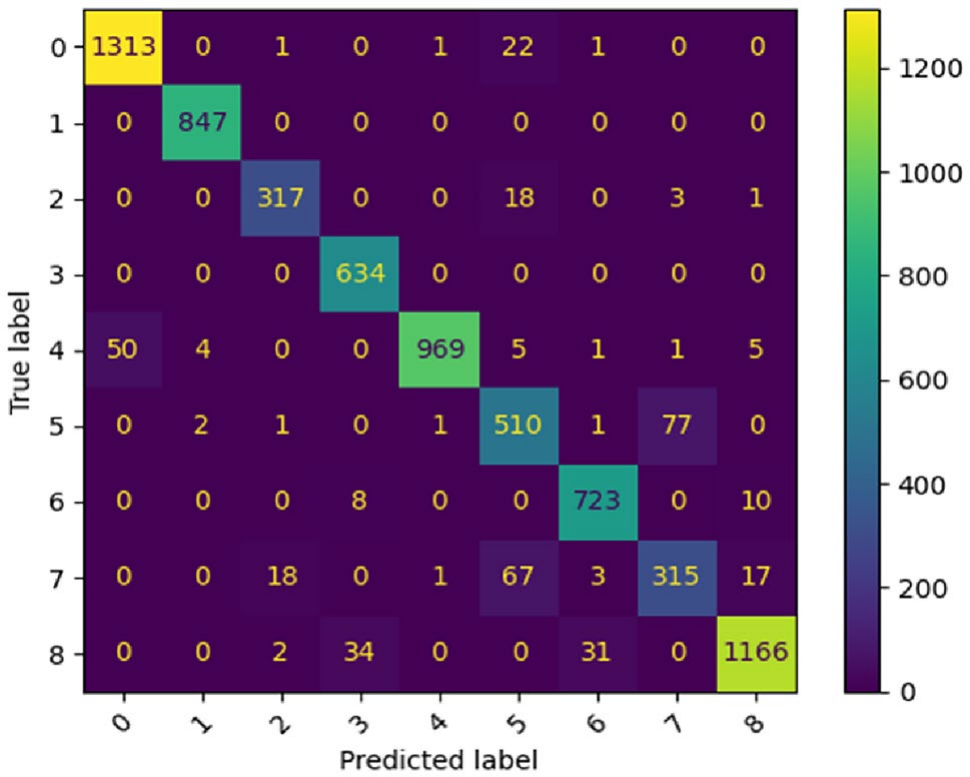
Confusion matrix produced by the proposed ViT model for PathMNIST dataset.

Table 5 shows the detailed classification report on the PathMNIST dataset. Figure 14 represents the confusion matrix for the PathMNIST dataset.

**Table 5:** Classification report (in terms of Precision, Recall, F1-Score and Support) given by the proposed ViT model of each class present in PathMNIST dataset.

Class label	Precision	Recall	F1-Score	Support
Adipose	0.96	0.98	0.97	1338
Background	1.00	1.00	1.00	847
Debris	0.94	0.94	0.94	339
Lymphocytes	0.94	1.00	0.97	634
Mucus	1.00	0.94	0.97	1035
Smooth muscle	0.82	0.86	0.84	592
Normal colon mucosa	0.95	0.98	0.96	741
Cancer-associated stroma	0.80	0.75	0.77	421
Colorectal adenocarcinoma epithelium	0.97	0.95	0.96	1233
ACC	94.62%

In case of Fig. 14, for ‘adipose’, the model correctly categorized 1313 images as ‘adipose’. The model misidentified one ‘adipose’ image as ‘debris’, one ‘adipose’ image as ‘mucus’, twenty two ‘adipose’ images as ‘smooth muscle’, one ‘adipose’ image as ‘normal colon mucosa’ and no ‘adipose’ image as rest of the classes. For ‘background’, the model correctly categorized 847 images as ‘background’. The model misclassified no ‘background’ images. For ‘debris’, the model correctly classified 317 images as ‘debris’. The model misclassified eighteen ‘debris’ images as ‘smooth muscle’, three ‘debris’ images as ‘cancer-associated stroma’, one ‘debris’ image as ‘colorectal adenocarcinoma epithelium’ and no ‘debris’ image as rest of the classes. For ‘lymphocytes’, the model correctly categorized 634 images as ‘lymphocytes’. The model misidentified no ‘lymphocytes’ images as rest of the classes. For ‘mucus’, the model correctly categorized 969 images as ‘mucus’. The model misclassified fifty ‘mucus’ images as ‘adipose’, four ‘mucus’ images as ‘background’, five ‘mucus’ images as ‘smooth muscle’, one ‘mucus’ image as ‘normal colon mucosa’, one ‘mucus’ image as ‘cancer-associated stroma’, five ‘mucus’ images as ‘colorectal adenocarcinoma epithelium’ and no ‘mucus’ image as rest of the classes. For ‘smooth muscle’, the model correctly categorized 510 images as ‘smooth muscle’. The model misidentified two ‘smooth muscle’ images as ‘background’ image, one ‘smooth muscle’ image as ‘debris’, one ‘smooth muscle’ image as ‘mucus’, one ‘smooth muscle’ image as ‘normal colon mucosa’, seventy seven ‘smooth muscle’ images as ‘cancer-associated stroma’ and no ‘smooth muscle’ image as rest of the classes. For ‘normal colon mucosa’, the model correctly classified 723 images as ‘normal colon mucosa’. The model misclassified eight ‘normal colon mucosa’ images as ‘lymphocytes’, ten ‘normal colon mucosa’ images as ‘colorectal adenocarcinoma epithelium’ and no ‘normal colon mucosa’ image as rest of the classes. For ‘cancer-associated stroma’, the model correctly classified 315 images as ‘cancer-associated stroma’. The model misclassified eighteen ‘cancer-associated stroma’ images as ‘debris’, one ‘cancer-associated stroma’ image as ‘mucus’, sixty seven ‘cancer-associated stroma’ images as ‘smooth muscle’, three ‘cancer-associated stroma’ images as ‘normal colon mucosa’, seventeen ‘cancer-associated stroma’ images as ‘colorectal adenocarcinoma epithelium’ and no ‘cancer-associated stroma’ image as rest of the classes. For ‘colorectal adenocarcinoma epithelium’, the model correctly classified 1166 images as ‘colorectal adenocarcinoma epithelium’. The model misclassified two ‘colorectal adenocarcinoma epithelium’ image as ‘debris’, thirty four ‘colorectal adenocarcinoma epithelium’ images as ‘lymphocytes’, thirty one ‘colorectal adenocarcinoma epithelium’ images as ‘normal colon mucosa’ and no ‘colorectal adenocarcinoma epithelium’ image as rest of the classes.

**Figure 15:**
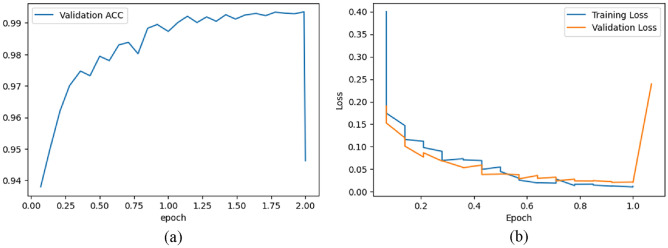
(**a**) Validation Accuracy & (**b**) Train-Validation loss graph for PathMNIST dataset.

Figure 15a represents the validation accuracy curve for the PathMNIST dataset over two epochs. In Fig. 15a, until slightly before around epoch = 2.00, the accuracy increases up to around 0.99 with fluctuations in between. Then the accuracy decreases steeply and at epoch = 2.00, the accuracy lies between 0.94 and 0.95. Figure 15b shows the training and validation loss of the model on the PathMNIST dataset over one epoch. The training loss decreases over time. The initial validation loss is between 0.15 and 0.20 which decreases until around epoch = 1. After epoch = 1, the validation loss increases.

**Figure 16:**
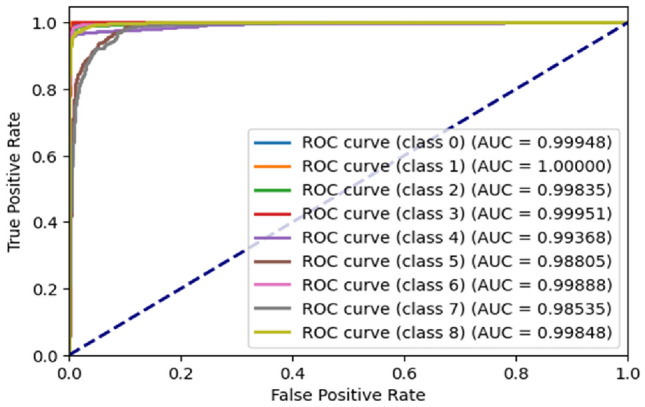
ROC curve generated by the proposed ViT model for PathMNIST dataset.

Figure 16 represents the ROC^21^ Curve for PathMNIST dataset. For class ‘0’, the AUC^21^ is 0.99948. For class ‘1’, the AUC is 1.00000 as no class ‘1’ image has been misclassified. For class ‘2’, the AUC is 0.99835. For class ‘3’, the AUC is 0.99951. For class ‘4’, the AUC is 0.99368. For class ‘5’, the AUC is 0.98805. For class ‘6’, the AUC is 0.99888. For class ‘7’, the AUC is 0.98535. For class ‘8’, the AUC is 0.99848.

### Results on RetinaMNIST

**Figure 17:**
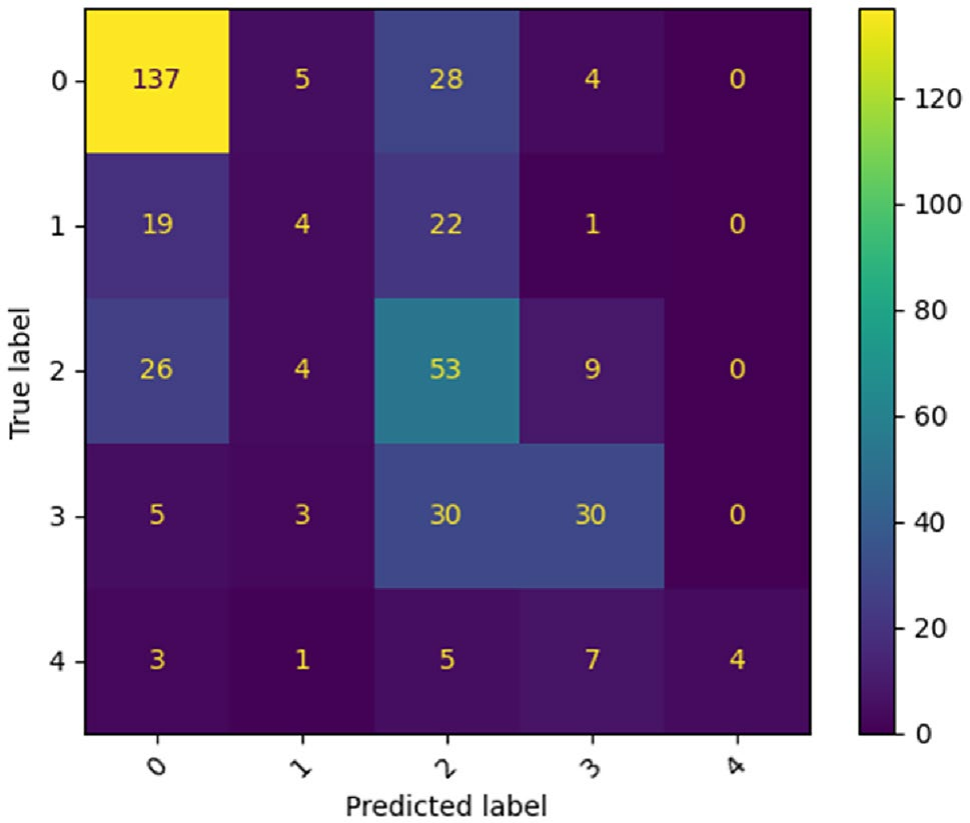
Confusion matrix produced by the proposed ViT model for RetinaMNIST dataset.

Table 6 shows the detailed classification report on the RetinaMNIST dataset. Figure 17 represents the confusion matrix for the RetinaMNIST dataset.

**Table 6:** Classification report (in terms of Precision, Recall, F1-Score and Support) given by the proposed ViT model of each class present in RetinaMNIST dataset.

Class label	Precision	Recall	F1-Score	Support
0	0.72	0.79	0.75	174
1	0.24	0.09	0.13	46
2	0.38	0.58	0.46	92
3	0.59	0.44	0.50	68
4	1.00	0.2	0.33	20
ACC	57.00%

In the case of Fig. 17, for class ‘0’, the model correctly classified 137 images as class ‘0’. The model misclassified five class ‘0’ images as class ‘1’, twenty eight class ‘0’ images as class ‘2’, four class ‘0’ images as class ‘3’ and no class ‘0’ images as rest of the classes. For class ‘1’, the model correctly classified 4 images as class ‘1’. The model misclassified nineteen class ‘1’ images as class ‘0’, twenty two class ‘1’ images as class ‘2’, one class ‘1’ images as class ‘3’ and no class ‘1’ images as rest of the classes. For class ‘2’, the model correctly categorized 53 images as class ‘2’. The model misidentified twenty six class ‘2’ images as class ‘0’, four class ‘2’ images as class ‘1’, nine class ‘2’ images as class ‘3’ and no class ‘2’ image as class ‘4’. For class ‘3’, the model correctly classified 30 images as class ‘3’. The model misclassified five class ‘3’ images as class ‘0’, three class ‘3’ images as class ‘1’, thirty class ‘3’ images as class ‘2’ and no class ‘3’ images as rest of the classes. For class ‘4’, the model correctly categorized 4 images as class ‘4’. The model misclassified three class ‘4’ images as class ‘0’, one class ‘4’ image as class ‘1’, five class ‘4’ images as class ‘2’ and seven class ‘4’ images as class ‘3’.

**Figure 18:**
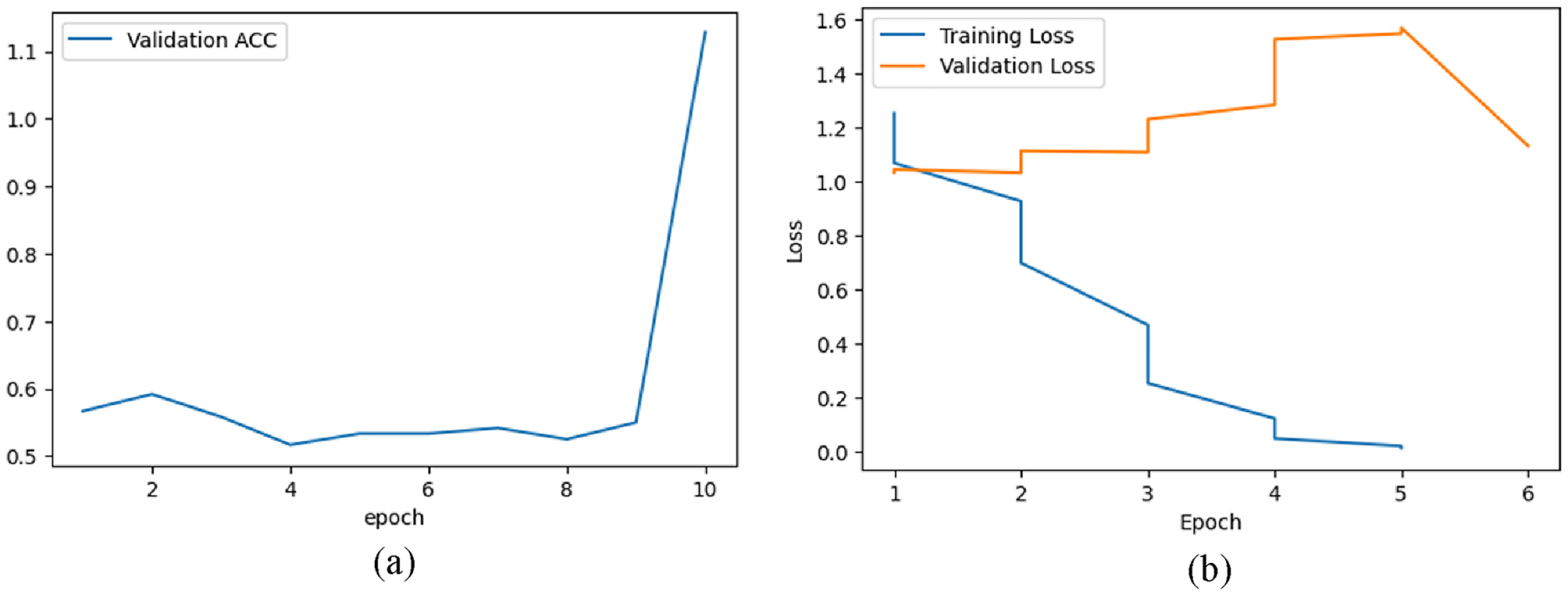
(**a**) Validation Accuracy & (**b**) Train-Validation Loss graph for RetinaMNIST dataset.

Figure 18a represents the validation accuracy curve for the RetinaMNIST dataset over ten epochs. From epoch = 0 to around epoch = 2, the validation accuracy increases to around 0.6. Then from epoch = 2 to epoch = 4, the accuracy decreases. From epoch = 8 to epoch = 10, the accuracy increases to around 1.1.

Figure 18b shows the training and validation loss of the model on the RetinaMNIST dataset over 6 epochs. The training loss decreases over time. The initial validation loss is around 1.0 and increases until epoch = 5 to around 1.6 with fluctuations in between. From epoch = 5 to epoch = 6, the validation loss decreases and at epoch = 6, the validation loss lies in between 1.0 and 1.2.

**Figure 19:**
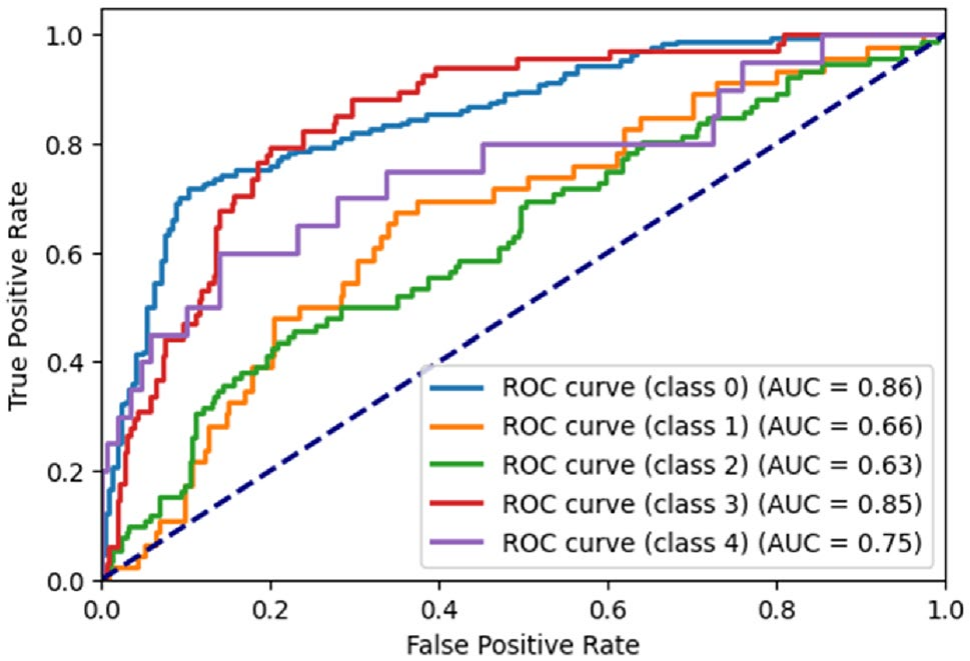
ROC curve generated by the proposed ViT model for RetinaMNIST dataset.

Figure 19 represents the ROC^21^ Curve for RetinaMNIST dataset. For class ‘0’, the AUC^21^ is 0.86. For class ‘1’, the AUC is 0.66. For class ‘2’, the AUC is 0.63. For class ‘3’, the AUC is 0.85. For class ‘4’, the AUC is 0.75.

### Grad-CAM analysis

Grad-CAM (Gradient Weighted Class Activation Mapping) is an interpretation technique used to visualise and understand the regions of an input that contribute most to the prediction made by a model particularly in the context of image classification tasks. It helps to identify which parts of the image are important for the model’s decision making process. In our study, the visualizations that have been generated through Grad-CAM have highlighted the model's attention to clinically relevant features, contributing to the robustness and explainability of our deep learning approach in medical image analysis. To use Grad-CAM and analyse the performance of our model on BloodMNIST, BreastMNIST, PathMNIST and RetinaMNIST dataset, we have used the package (https://github.com/jacobgil/pytorch-grad-cam) which is publicly available on Github. We have performed a reshape transform which is specific to the ViT model. It rearranges activations from the model's output to prepare them for Grad-CAM processing. The ViT model's output typically includes class logits and other information, and this function extracts relevant information for Grad-CAM.

**Figure 20:**
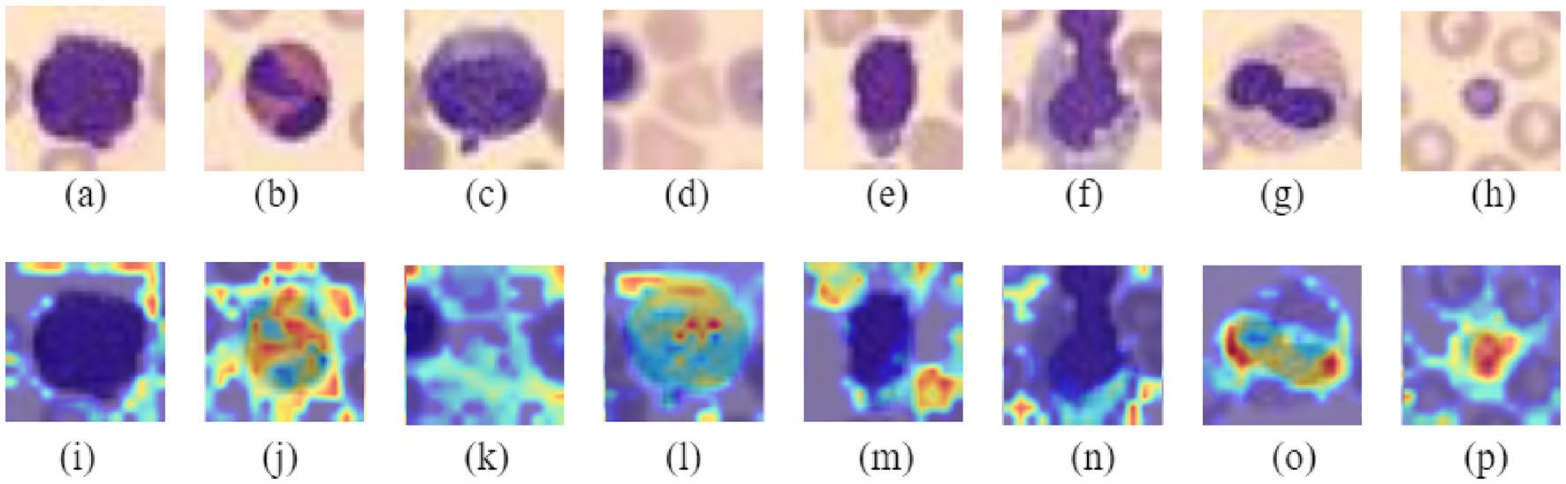
Various classes of BloodMNIST dataset shown in (**a**–**h**) and their corresponding Grad-CAM visualization images illustrated in (**i**–**p**).

**Figure 21:**
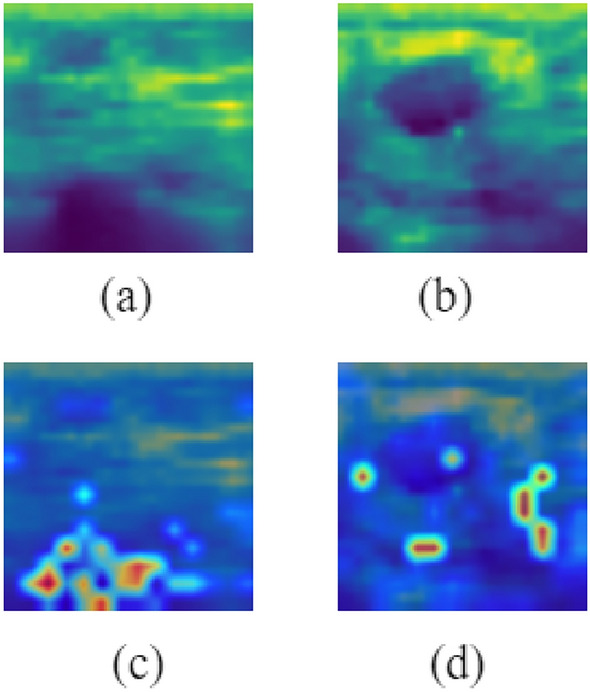
Various classes of BreastMNIST dataset shown in (**a**, **b**) whereas (**c**, **d**) illustrates their corresponding Grad-CAM visualization images.

**Figure 22:**
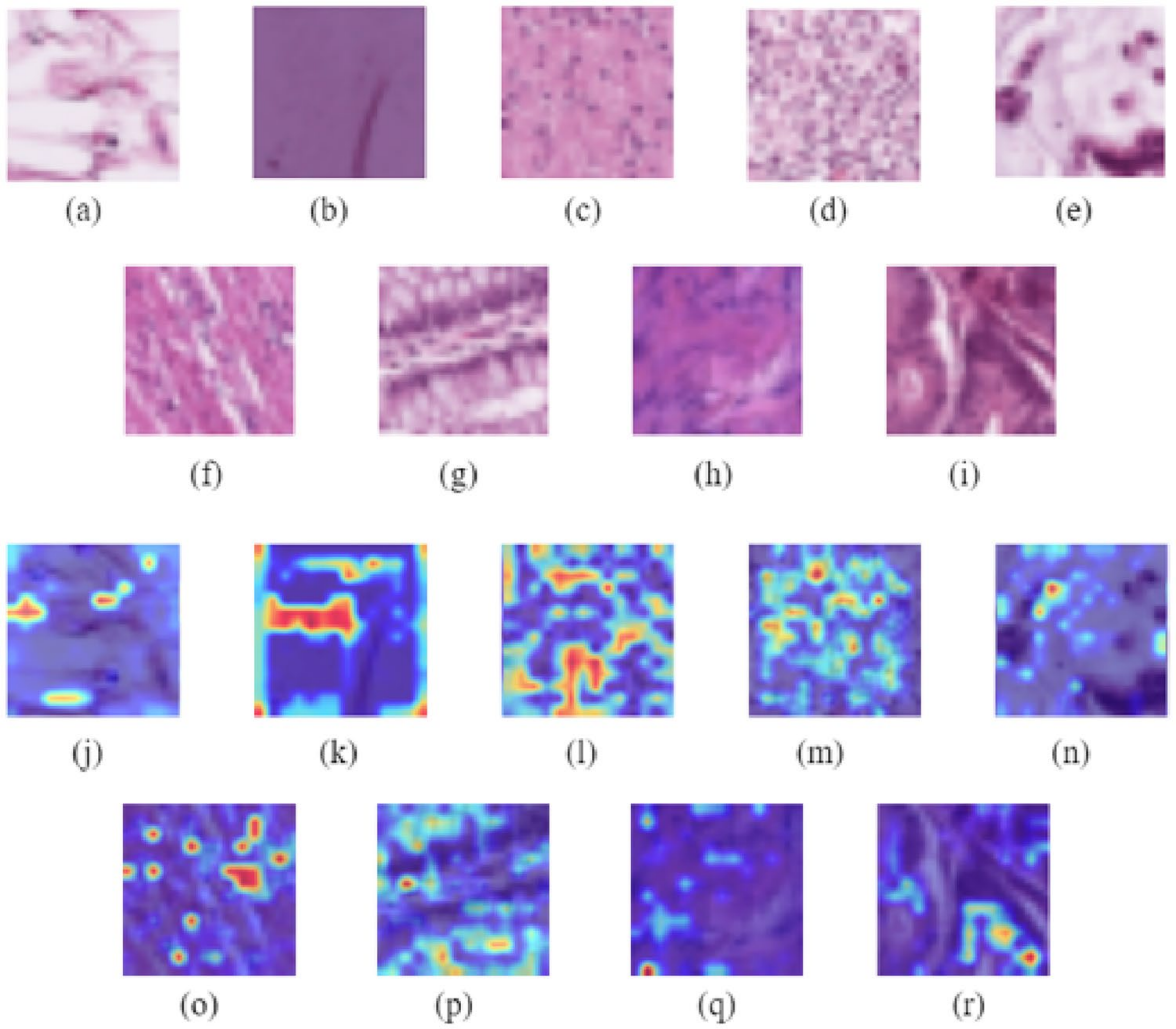
Various classes of PathMNIST dataset as shown in (**a**–**i**) and their corresponding Grad-CAM visualization images illustrated in (**j**–**r**).

**Figure 23:**
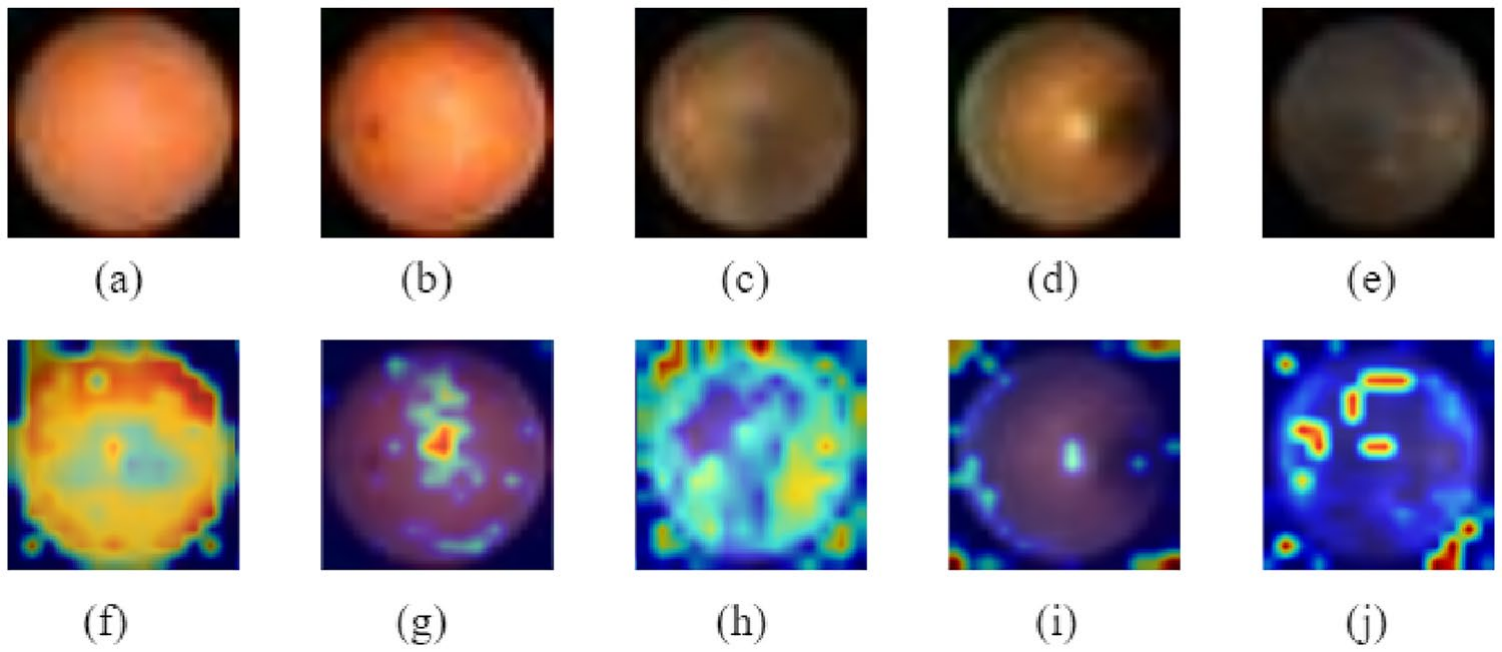
Various classes of RetinaMNIST dataset and their corresponding Grad-CAM visualization images.

Figure 20 shows the Grad-CAM analysis on BloodMNIST dataset. It shows the original images of each of the eight classes (‘basophil’, ‘eosinophil’, ‘erythroblast’, ‘immature granulocytes’, ‘lymphocyte’, ‘monocyte’, ‘neutrophil’, ‘platelet’) and their corresponding heatmaps which highlight the parts that have been relevant for making predictions. Figure 21 shows the Grad-CAM analysis on BreastMNIST dataset. It shows the original images of each of the three classes (‘malignant’, ‘normal, benign’) and their corresponding heatmaps which highlight the parts that have been relevant for making predictions. Figure 22 shows the Grad-CAM analysis on the PathMNIST dataset. It shows the original images of each of the classes and their corresponding heatmaps which highlight the parts that have been relevant for making predictions. Figure 23 shows the Grad-CAM analysis on the RetinaMNIST dataset. It shows the original images of each of the classes and their corresponding heatmaps which highlight the parts that have been relevant for making predictions.

### Comparison with benchmark approaches

Table 7 shows the comparison of the accuracy achieved by the fine-tuned ViT-Base-Patch16-224 model on the BloodMNIST, BreastMNIST, PathMNIST, RetinaMNIST datasets against the benchmark accuracies for the respective datasets. The benchmark accuracies are also available in the MedMNISTv2 documentation^1^. For the BloodMNIST dataset, our model achieves an accuracy of 97.90% beating the benchmark accuracy of 96.6% which is achieved by Google AutoML Vision^33^. For the BreastMNIST dataset, our model achieves an accuracy of 90.38% beating the benchmark accuracy of 86.3% which is achieved by ResNet-18^5^ (28). For the PathMNIST dataset, our model achieves an accuracy of 94.62% beating the benchmark accuracy of 91.1% which is achieved by ResNet-50^5^ (28). For the RetinaMNIST dataset, our model achieves an accuracy of 57.0% beating the benchmark accuracy of 53.1% which is achieved by Google AutoML Vision^33^.

**Table 7:** Comparison of our approach for BloodMNIST, BreastMNIST, PathMNIST and RetinaMNIST dataset against some standard model architectures.

Models	BloodMNIST (%)	BreastMNIST (%)	PathMNIST (%)	RetinaMNIST (%)
ResNet-18 (28)	95.8	86.3	90.7	52.4
ResNet-18 (224)	96.3	83.3	90.9	49.3
ResNet-50 (28)	95.6	81.2	91.1	52.8
ResNet-50 (224)	95.0	84.2	89.2	51.1
auto-sklearn	87.8	80.3	71.6	51.5
AutoKeras	96.1	83.1	83.4	50.3
Google AutoML Vision	96.6	86.1	72.8	53.1
ViT-Base-Patch16-224	97.90	90.38	94.62	57.0

## Conclusion & future works

In this study, we explored the application of ViT-Base-Patch16–224 model for the classification of diverse 2D biomedical image datasets namely BloodMNIST, BreastMNIST, PathMNIST, RetinaMNIST and the model proved highly promising in the classification process. Our proposed model has not only achieved impressive accuracies surpassing all existing benchmarks but has also showcased robust adaptability and performance. With achieved classification accuracies of BloodMNIST: 97.90%, BreastMNIST: 90.38%, PathMNIST: 94.62%, RetinaMNIST: 57%, our findings underscore the transformative potential of our proposed model in revolutionising biomedical image classification, suggesting opportunities for significantly enhancing diagnostic workflows in healthcare.

Our proposed model has shown outstanding performance and achieved accuracies surpassing all existing benchmarks. However, our pursuit for advancement remains relentless. In the future, leveraging high-spec GPU devices, we can increase the number of patches derived from the images within each class and potentially enrich the dataset’s representation and feature diversity. Expanding the diversity and scope of biomedical image datasets plays a pivotal role in contributing to a more comprehensive understanding of varied medical conditions. Furthermore, the adoption of explainable AI techniques stands as a critical factor in the integration of AI into healthcare practices.

## Experiment

All experiments and methods were carried out in accordance with relevant guidelines and regulations. All experimental protocols were approved by Department of Information Technology, Jadavpur University, Kolkata, India. Informed consent was obtained from all subjects and/or their legal guardian(s).

## Data Availability

No datasets are generated during the current study. The datasets analyzed during this work can be found at: https://medmnist.com/.

## Code availability

All the source codes related to this research work can be found at: https://github.com/arindam369/MedMNIST-ViT

## References

[1] Yang J, Shi R, Wei D (2023). MedMNIST v2 - A large-scale lightweight benchmark for 2D and 3D biomedical image classification. Sci. Data.

[2] Ghalati MK, Nunes A, Ferreira H, Serranho P, Bernardes R (2022). Texture analysis and its applications in biomedical imaging: A survey. IEEE Rev. Biomed. Eng..

[3] Dosovitskiy, A., Beyer, L., Kolesnikov, A., Weissenborn, D., Zhai, X., Unterthiner, T., Dehghani, M., Minderer, M., Heigold, G., Gelly, S., Uszkoreit, J., Houlsby, N. An image is worth 16x16 words: Transformers for image recognition at scale. https://arxiv.org/abs/2010.11929 (2020)

[4] Sultana, F., Sufian, A., Dutta, P. Advancements in image classification using convolutional neural network. In *2018 Fourth International Conference on Research in Computational Intelligence and Communication Networks (ICRCICN)*, Kolkata, India 122–129 (2018) 10.1109/ICRCICN.2018.8718718.

[5] Khan, R. U., Zhang, X., Kumar, R., Aboagye, E. O. Evaluating the performance of ResNet model based on image recognition. In *Proceedings of the 2018 International Conference on Computing and Artificial Intelligence (ICCAI '18)*. Association for Computing Machinery, New York 86–90 (2018) 10.1145/3194452.3194461

[6] Abai, Z., & Rajmalwar, N. Densenet models for tiny imagenet classification. arXiv preprint https://arxiv.org/abs/1904.10429 (2019).

[7] Ridnik, T., Ben-Baruch, E., Noy, A., & Zelnik-Manor, L. Imagenet-21k pretraining for the masses. arXiv preprint https://arxiv.org/abs/2104.10972 (2021).

[8] Russakovsky O, Deng J, Su H (2015). ImageNet large scale visual recognition challenge. Int. J. Comput. Vis..

[9] ViT-Base-Patch16-224 Model: https://huggingface.co/google/vit-base-patch16-224

[10] Yang, J., Shi, R., & Ni, B. MedMNIST classification decathlon: A lightweight AutoML benchmark for medical image analysis. In *2021 IEEE 18th International Symposium on Biomedical Imaging (ISBI)*, Nice, France, 191–195 (2021). 10.1109/ISBI48211.2021.9434062.

[11] He X, Zhao K, Chu X (2021). AutoML: A survey of the state-of-the-art. Knowl. -Based Syst..

[12] Jin, H., Song, Q., & Hu, X. Auto-Keras: An efficient neural architecture search system. In *Proceedings of the 25th ACM SIGKDD International Conference on Knowledge Discovery & Data Mining (KDD '19)*. Association for Computing Machinery, New York 1946–1956 (2019). 10.1145/3292500.3330648

[13] Liu, J., Li, Y., Cao, G., Liu, Y., & Cao, W. Feature pyramid vision transformer for MedMNIST classification decathlon. In *2022 International Joint Conference on Neural Networks (IJCNN)*, Padua, Italy, 1–8 (2022) 10.1109/IJCNN55064.2022.9892282.

[14] Lu, C., & Kalpathy-Cramer, J. Distribution-free federated learning with conformal predictions. arXiv preprint https://arxiv.org/abs/2110.07661 (2021).

[15] Nejati Manzari, O., Ahmadabadi, H., Kashiani, H., Shokouhi, S., Ayatollahi, A. (2023). MedViT: A robust vision transformer for generalized medical image classification. 10.48550/arXiv.2302.09462. 10.1016/j.compbiomed.2023.106791 36958234

[16] Khan, P.I., Dengel, A. and Ahmed, S. Medi-CAT: Contrastive adversarial training for medical image classification. arXiv preprint https://arxiv.org/abs/2311.00154 (2023)

[17] Saha, P., Mishra, D., & Noble, J. Rethinking semi-supervised federated learning: How to co-train fully-labelled and fully-unlabeled client imaging data. 10.1007/978-3-031-43895-0_39. (2023)

[18] Herrmann, C., Sargent, K., Jiang, L., Zabih, R., Chang, H., Liu, C., Krishnan, D., & Sun, D. Pyramid adversarial training improves ViT performance*. In 2022 IEEE/CVF Conference on Computer Vision and Pattern Recognition (CVPR)*, 13409–13419. 10.48550/arXiv.2111.15121 (2021).

[19] ImageNet-1k Dataset: https://huggingface.co/datasets/imagenet-1k

[20] Nguyen, N. -Q., & Le, T. -S. A semi-supervised learning method to remedy the lack of labeled data. In *2021 15th International Conference on Advanced Computing and Applications (ACOMP)*, Ho Chi Minh City, Vietnam 78–84 (2021) 10.1109/ACOMP53746.2021.00017.

[21] Bradley AP (1997). The use of the area under the ROC curve in the evaluation of machine learning algorithms. Pattern Recogn..

[22] Xu M, Zhang T, Zhang D (2022). MedRDF: a robust and retrain-less diagnostic framework for medical pretrained models against adversarial attack. IEEE Trans. Med. Imaging.

[23] Acevedo A, Merino A, Alférez S, Molina Á, Boldú L, Rodellar J (2020). A dataset of microscopic peripheral blood cell images for development of automatic recognition systems. Data Brief.

[24] Acevedo A (2020). A dataset for microscopic peripheral blood cell images for development of automatic recognition systems. Mendeley Data.

[25] Al-Dhabyani W, Gomaa M, Khaled H, Fahmy A (2020). Dataset of breast ultrasound images. Data Brief.

[26] Kather JN, Krisam J, Charoentong P, Luedde T, Herpel E, Weis CA, Gaiser T (2019). Predicting survival from colorectal cancer histology slides using deep learning: A retrospective multicenter study. PLoS Med..

[27] Kather JN, Halama N, Marx A (2018). Zenodo.

[28] Liu R, Wang X, Wu Q, Dai L, Fang X, Yan T, Son J, Tang S, Li J, Gao Z, Galdran A, Poorneshwaran JM, Liu H, Wang J, Chen Y, Porwal P, Tan GSW, Yang X, Dai C, Song H, Chen M, Li H, Jia W, Shen D, Sheng B, Zhang P (2022). DeepDRiD: Diabetic retinopathy—grading and image quality estimation challenge. Patterns.

[29] Google Vision Transformer Repository: https://github.com/google-research/vision_transformer

[30] Bebis G, Georgiopoulos M (1994). Feed-forward neural networks. IEEE Potentials.

[31] Loshchilov, I., & Hutter, F. Decoupled weight decay regularization. arXiv preprint https://arxiv.org/abs/1711.05101 (2017).

[32] Yacouby, R., & Axman, D. Probabilistic extension of precision, recall, and F1 score for more thorough evaluation of classification models. 79–91. 10.18653/v1/2020.eval4nlp-1.9 (2020).

[33] Google AutoML Vision: https://cloud.google.com/vision/automl/docs

